# Dynamic modelling of feed assimilation, growth, lipid accumulation, and CO_2_ production in black soldier fly larvae

**DOI:** 10.1371/journal.pone.0276605

**Published:** 2022-10-26

**Authors:** Niels Thomas Eriksen

**Affiliations:** Department of Chemistry and Bioscience, Aalborg University, Aalborg, Denmark; University of Illinois, UNITED STATES

## Abstract

The black soldier fly (BSF) is becoming a novel farm animal. BSF larvae can be reared on different substrates. Their performance is important but highly variable and different models have been employed to analyze their growth, so far without considering that metabolic rates, growth, and biochemical composition of the larvae are interrelated. This work develops a dynamic model, which describes general growth patterns of BSF larvae and predicts observed variability in larval performances. The model was tested against data from literature, which combines kinetic growth data with measurements of lipid or dry weight content, and CO_2_ production. The model combines the kinetics of the logistic model with principles from differential energy budget models and considers key events in larval life history, moulting and metamorphosis. Larvae are compartmentised into structural biomass, storage lipids, and a pool of assimilates. Feed assimilation is considered the overall rate limiting process and is reduced in relation to larval weight by a logistic function. A second logistic function further reduces the specific growth rate of structural biomass, causes imbalance between and feed assimilation and growth rates, and leaves a surplus of assimilates to be stored as lipids. Fluxes between compartments consider cost of synthesis of structural biomass and lipids, as well as maintenance. When assimilation falls below maintenance needs, storage lipids are recycled. The model is able to describe growth and lipid contents of BSF larvae reared on chicken feed, growth of feed limited BSF larvae, as well as growth, dry weight content, and CO_2_ production of BSF larvae reared on different substrate qualities and moisture contents. The model may be used for the analysis of growth and performance of BSF larvae under variable rearing conditions. It can deepen the analyses of experimental data and provide insight into the causes of variability of larval performances.

## Introduction

Larvae of the black soldier fly (BSF, *Hermetia illucens*) are promising candidates for upgrading of organic materials into high quality biomass [1] for use in e.g., animal feed [2] or biodiesel [3], and for reduction of organic waste streams [4–6]. BSF larvae can be reared on a variety of substrates, including organic wastes, sludge, manure, agricultural by-products, and commercial animal feed blends. Larval performances, in terms of growth, size, biochemical composition, and life span, as well as the efficiency by which substrates are converted into larval biomass vary considerably and are affected by the quality of the substrate [1, 7–9], substrate supply [10], as well as temperature, oxygen availability, and moisture content [11–14]. Larval performances are difficult to compare across studies due to the complexity of the substrates and variations of the physical conditions within and between larval rearing cultures.

Growth and development rely on complex processes and growth models are essential tools for the analysis and quantification of animal growth. It has become increasingly popular to apply growth models for the analysis of growth and development of BSF larvae in recent years. Growth of BSF larvae have been described by sigmoidal Gompertz models [15, 16] and logistic Richards and Verhulst models [17]. The logistic models have been found to describe growth of BSF larvae (in terms of increasing body width) most accurately and used to evaluate the quality of different substrates [17]. Verhulst’s logistic model has also been used to estimate specific growth rates and maximal weight of BSF larvae, which combined with measured respiration rates also enabled costs of growth, maintenance, and net growth efficiency to be quantified [9, 14]. Probably the most comprehensive growth model for BSF larvae describes growth as a function of feed assimilation [18]. In this model, a logistic function decreases the feed assimilation rate in relation to the increasing weight of the larvae. The growth rate is determined by the difference between the rate of ingestion and the rates of excretion and metabolic processes, and growth slows down as the larvae approach their maximal weight. The feed assimilation rate was furthermore modelled as a function of limited feed availability, temperature, oxygen availability, or moisture content. One alternative growth model for BSF larvae, not based on logistic functions, was developed by [19]. This model links growth at the culture level to overall substrate reduction but makes little use of physiological data.

The biochemical composition of insect larvae varies over their life span, not least their lipid content. Increasing amounts of storage lipids accumulate in the fat body as the larvae become larger, and these can later be re-mobilized to supply energy [20]. This is the case also for BSF larvae, which become increasingly rich in lipids as they grow but loose again most of their lipid reserves when they become prepupae [21–23]. The accumulation of lipids in the fat body of BSF larvae depends, however, on their dietary status [24]. If growth is slow and the weight of the prepupae low, or when nutritionally important substrate components are spent before the larvae are fully grown, the prepupae end up with low lipid contents [25–27]. Lipid contents of BSF larvae and prepupae at time of harvest may thus vary between 8% and 47% per unit of dry biomass [28]. The water content in the larval tissue will presumably show an inverse relationship to lipid content since adipose tissues binds relatively little water [29], and fluctuations in water content have also been observed in growing BSF larvae [9, 14]. These size and age dependent differences in biochemical composition shows that rates of feed uptake and growth are not balanced in growing BSF larvae, something that is not considered in the growth models described above [9, 14–18].

A general model for growth of BSF larvae should be able to describe general growth patterns and be sufficiently dynamic to also encompass the variability in growth rates and larval weights that are observed when BSF larvae are reared on different substrates and at different conditions. The weight of the larvae represents the combined weight of all the components that form their body, but biochemical composition is unaccounted for in the growth models described above, despite apparent links between growth, metabolism, and lipid accumulation. The aim of this paper is therefore to develop a dynamic model for BSF larvae, which combines the kinetics of the logistic model with principles from differential energy budget (DEB) models, which structure the biomass into compartments and predict metabolite fluxes and biochemical composition, across a range of substrates and environmental conditions. Such a model may improve and deepen the analyses of data from growth experiments, provide insight into the overall rates of anabolic and catabolic processes responsible for growth and development of BSF larvae, and be used for optimization of the conditions in rearing cultures of BSF larvae.

## Growth model development

**Fig 1:**
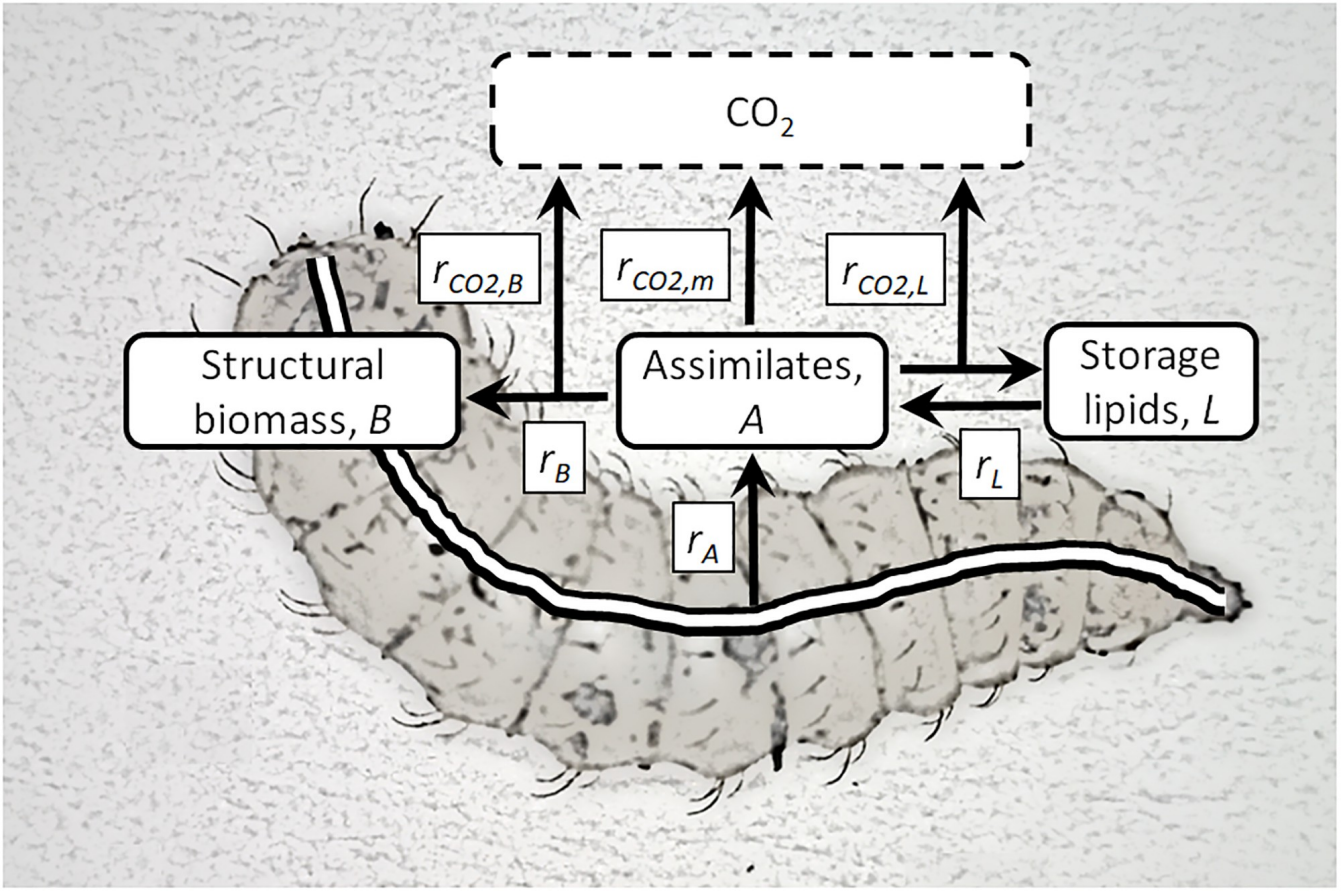
Differential energy budget model of BSF larva. Larval biomass is compartmentised into structural biomass, *B*, storage lipids, *L*, and a pool of assimilates, *A*. Feed components are assimilated (from the intestine) into the pool of assimilates at rate, *r*_*A*_ from where they are metabolized to CO_2_ to generate energy for maintenance at rate *r*_*CO2*,*m*_, utilized for structural biomass or converted into storage lipids at rates *r*_*B*_ and *r*_*L*_, or metabolized to CO_2_ to generate energy for the synthesis of structural biomass or storage lipids at rates *r*_*CO2*,*B*_ and *r*_*CO2*,*L*_. Storage lipids can be recycled to the pool of assimilates.

The growth model developed in this study combines the kinetics of the logistic function with a DEB model where larval biomass is compartmentised into structural biomass, *B* and storage lipids, *L*. In the model, the structural biomass includes all components of the larvae besides the lipids. Feed components are assumed to enter a pool of assimilates, *A* from where they are utilized for production of structural biomass, metabolized to CO_2_ to generate energy for maintenance, or converted into storage lipids. Synthesis of structural biomass and lipids (observable as growth) are energy demanding anabolic processes and parts of the assimilates allocated for structural biomass or storage lipids are metabolized to CO_2_ via catabolism. In the model, maintenance covers all energy demanding processes that do not lead to net synthesis of structural biomass or lipids. Storage lipids can be recycled to the pool of assimilates. In the model, the pool of assimilates is thus considered to be molecules in transit from they are released into the hemolymph from either the intestinal wall or the fat body and until they undergo energy demanding biochemical modifications. The main structural components and overall metabolic processes are illustrated in Fig 1.

The pool of assimilates has probably never been quantified in BSF larvae but is likely small in mass compared to the masses of structural biomass and storage lipids. Potential changes in the mass of assimilates will therefore also be small compared to the fluxes of assimilates passing through. In the model, the pool of assimilates is therefore assumed to be at steady-state. Mass balances of assimilates, structural biomass, and lipids are shown in Eqs 1–3.

$$\frac{dA}{dt}=0=r_{A}-\left(r_{B}+r_{L}+r_{CO2,m}+r_{CO2,B}+r_{CO2,L}\right)$$ (1)

$$\frac{dB}{dt}=r_{B}$$ (2)

$$\frac{dL_{tot}}{dt}=r_{L}+\delta_{L,B}r_{B}$$ (3)

where *r*_*A*_, *r*_*B*_, and *r*_*L*_ are rates of feed assimilation, synthesis of structural biomass, and accumulation of storage lipids, while *r*_*CO2*,*m*_, *r*_*CO2*,*B*_, and *r*_*CO2*,*L*_ are rates of CO_2_ production due to energy requirements associated with maintenance, synthesis of structural biomass, and synthesis of storage lipids, respectively. Lipids also make up a fraction, *δ*_*L*,*B*_ of the structural biomass which are analytically indistinguishable from storage lipids, and *L*_*tot*_ represents the total lipid content in a larva, i.e. the amount of lipid that can be measured experimentally.

The feed assimilation rate is considered the overall rate limiting metabolic process, expressed as the product of the specific feed assimilation rate, *a* and the weight of structural biomass.


$$r_{A}=aB$$ (4)

Insect larvae show a discontinuous mode of growth where moulting phases separate the different instars, *I*. The life history of BSF larvae includes 6 instars before they reach maximal size, turn into prepupae, and stop feeding [30]. A 7^th^ instar was also described by [31], which do not grow and here is viewed as part the prepupal stage. When conditions are suitable, BSF larvae grow exponentially until they reach a certain weight [9, 14]. The transition from instar 5 to instar 6 marks the end of exponential growth, and when the larvae turn into pupae growth ceases. This growth pattern is due to differences in specific feed uptake rate in the different instars, in the model described by Eq 5.

$$a=\left\{\begin{array}{ll} a_{max}, & I<6\\ a_{max}\left(1-{\left(\frac{B}{B_{max}}\right)}^{\alpha }\right), & I=6\\ 0, & I>6 \end{array}\right.$$ (5)

The maximal specific feed assimilation rate, *a*_*max*_ is sustained by instar 1–5. The specific feed assimilation rate decreases in instar 6, following a logistic expression where *B*_*max*_ is the maximal weight of structural biomass of a BSF larva. The coefficient, *α* adjusts how rapidly assimilation slows down with increasing structural biomass weight. If *α* = 1, the expression is the same as in Verhulst’s logistic model, used also in the growth model by [18]. If *α* is smaller or larger than 1, the decrease in specific assimilation rate is accelerated or decelerated as compared to Verhulst’s logistic model. When the larvae transform into prepupae they stop feeding. This pattern of feed uptake is reflected also in the metabolic rates of BSF larvae. Instars 3 and 4 are characterized by a high specific heat production rate, which slows down in instars 5 and 6, to reach minimum in instar 7 [31].

Part of the assimilated feed is metabolized to generate energy for maintenance. Maintenance is considered to be proportional to the mass of structural biomass [32] and the maintenance rate, expressed in terms of CO_2_ production, is described by Eq 6

$$r_{CO2,m}=mB$$ (6)

where *m* is the specific rate of CO_2_ production due to maintenance metabolism.

The growth rate of structural biomass will depend on the specific growth rate of this component, *μ*_*B*_. When the larvae turn into prepupae, the specific growth rate becomes zero and no more structural biomass is produced.

$$r_{B}=\left\{\begin{array}{ll} \mu_{B}B, & I\leq 6\\ 0, & I>6 \end{array}\right.$$ (7)

If assimilation and growth are balanced, storage lipids will not accumulate, the specific growth rate will correspond to the difference between specific rates of assimilation and respiratory losses and represent the potential for structural growth

$$\mu_{B}=a-q$$ (8)

where *q* is the specific rate of CO_2_ production. Assimilates are respired to CO_2_ to supply energy for growth as well as for maintenance

$$q=Y_{B}\mu_{B}+m$$ (9)

where *Y*_*B*_ (dimensionless) represents the cost of growth of structural biomass and depends on the energetic needs for conversion of feed into structural biomass. Growth and feed assimilation is, however, not balanced in BSF larvae because their biochemical composition is size dependent, and their lipid content increases with age [21–23]. This can be interpreted as if late instars produce structural biomass at a lower specific rate than predicted by Eq 8. The specific growth rate is therefore modelled by Eq 10, in which Eqs 8 and 9 are combined to predict the potential for growth of structural biomass (expression within the first parenthesis) multiplied by a second logistic expression (expression within the second parenthesis), forcing the specific growth rate further and further below its potential as the larvae approach maximal weight.

$$\mu_{B}=\left(\frac{a-m}{1+Y_{B}}\right)\left(1-{\left(\frac{B}{B_{max}}\right)}^{\beta }\right)$$ (10)

The coefficient, *β* adjusts how rapidly the specific growth rate is decreasing with body weight, in addition to the decrease mediated via the decreasing specific assimilation rate. The smaller *β* is, the larger will the fraction of assimilates, which are guided towards storage lipids, be.

The rate of CO_2_ production due to the energy metabolism associated with production of structural biomass is estimated from Eq 11.


$$r_{CO2,B}=Y_{B}r_{B}$$ (11)

Based on the steady-state assumption in Eq 1, accumulation of storage lipids can now be calculated as the difference between the assimilation rate and the processes accounted for in Eqs 4, 6, and 7. In case assimilates are in surplus, these are used for storage lipid synthesis. If not, stored lipids will be returned to the pool of assimilates

$$r_{L}=\left\{\begin{array}{ll} \frac{r_{a}-\left(r_{B}+r_{CO2,m}+r_{CO2,B}\right)}{1+Y_{L}}, & r_{L}\geq 0\\ r_{a}-\left(r_{B}+r_{CO2,m}+r_{CO2,B}\right), & r_{L}<0 \end{array}\right.$$ (12)

Lipid synthesis takes place via energy demanding biosynthesis processes. The rate of CO_2_ production stemming from storage lipid synthesis is calculated from Eq 13

$$r_{CO2,L}=\left\{\begin{array}{ll} Y_{L}r_{L}, & r_{L}\geq 0\\ 0, & r_{L}<0 \end{array}\right.$$ (13)

where *Y*_*L*_ represents the cost of converting assimilates into lipids.

The overall CO_2_ production rate is calculated as the sum of the 3 CO_2_ producing processes described by Eqs 6, 11, and 13

$$r_{CO2}=r_{CO2,m}+r_{CO2,B}+r_{CO2,L}$$ (14)

In order to use Eqs 5 and 7, the instar number must be predicted. Insect larvae grow to critical weights at which they initiate a hormone response that gradually terminates feeding and growth and leads to metamorphosis [33]. In fruit flies, the critical weight seems unaffected by nutritional conditions while slow growth prolongs the periods in between moulting phases. Similar hormone responses also initiate the moulting phases [33] and the relative increase in weight is about the same for each instar [34]. The critical weight is hardly ever identified in studies on BSF larvae. Instead, the instar numbers can be related to the maximal weight of structural biomass,

$$I=\left\{\begin{array}{ll} 1-5, & B<\frac{1}{\delta_{I}}B_{max,0}\\ 6, & \frac{1}{\delta_{I}}B_{max,0}\leq B<B_{max,0}\\ 7, & B\geq B_{max,0} \end{array}\right.$$ (15)

where *δ*_*I*_ is the weight ratio between two succeeding moulting phases, and *B*_*max*,*0*_ is the maximal weight of structural biomass under optimal growth conditions.

BSF larvae grow to variable weights. Instar 6 is responsible for most of the weight gain and low final weight is related to slow growth and prolonged developmental time of instar 6 [10]. The same is seen in fruit flies, in which the final instar may increase in weight more than 4 times after the critical weight is reached while physical and dietary conditions affect their growth rate as well as their final weight [33]. In order to take into account the negative relationship between final weight and developmental time, the maximal weight of structural biomass is described as a time dependent variable, which decreases linearly (the simplest possible function) with time when the larvae have not developed into prepupae after a certain time, *t*_*p*,*min*_, which is the shortest time needed for BSF larvae to reach the prepupal stage.

$$B_{max}=\left\{\begin{array}{ll} B_{max,0}, & t<t_{p,min}\\ B_{max,0}-\rho \left(t-t_{p,min}\right), & t\geq t_{p,min} \end{array}\right.$$ (16)

The parameter, *ρ* describes the daily rate by which the maximal weight of structural biomass then decreases.

The parameters and variables in Eqs 1–14 and 16 were expressed in terms of carbon equivalents by multiplication with the carbon content of structural biomass, the carbon content of lipids, or the carbon content of CO_2_. Thereby, each term in the model was expressed in the same unit and made additive.

## Methods

### Simulation of growth curves and lipid contents

To simulate growth curves and changes in biomass composition, Eqs 2 and 3 were solved numerically using Euler’s method

$$B_{t}=B_{t+\Delta t}+r_{B,t}\Delta t$$ (17)

$$L_{tot,t}=L_{tot,t+\Delta t}+\left(r_{L,t}+\delta_{L}r_{B,t}\right)\Delta t$$ (18)

at time intervals, *Δt* = 0.01–0.02 day. The subscripts, *t* and *t+Δt* symbolize start- and end-values of each time interval. Eqs 17 and 18 were programmed into Excel in 2,000 time intervals, each represented by a row in the spreadsheet. The rates of feed assimilation, maintenance, formation of structural biomass, storage lipids, and CO_2_ were subsequently calculated for each time interval using Eqs 4–6 and 10–12, and so the instar number and the maximal weight of structural biomass (Eqs 15 and 16). The model is available as S1 Data.

### Estimation of larval dry weight and weight of lipids

To compare results of the model to experimental data available in the literature, structural biomass and lipid contents, calculated from Eqs 17 and 18, were converted from carbon equivalents to larval dry weight, DW by Eq 19, which takes into account the carbon content in the structural biomass as well as in the structural lipids, and that the structural biomass contains a fraction of inorganic components.

$$X_{DW}=\frac{\left(1-\delta_{L,B}\right)B}{\delta_{C,B}\delta_{O}}+\frac{L_{tot}}{\delta_{C,L}}$$ (19)

*X*_*DW*_ is larval DW and *δ*_*C*,*B*_ and *δ*_*C*,*L*_ are the fractions of carbon in the organic part of the structural biomass, and in the lipids, respectively, and *δ*_*O*_ is the organic part of the structural biomass. Modelled lipid contents were converted to lipid DW by Eq 20.

$$L_{DW}=\frac{L_{tot}}{\delta_{C,L}}$$ (20)

and the overall lipid fraction of the larval biomass DW, *δ*_*lipid*_ was calculated from Eq 21

$$\delta_{lipid}=\frac{L_{DW}}{X_{DW}}$$ (21)

Lipids bind water poorly and high lipid contents will lower the water content and increase the DW content of BSF larvae. This effect was taken into account in Eq 22, which predicts the DW fraction, *δ*_*DW*_ of the larval wet weight, WW

$$\delta_{DW}=\frac{X_{DW}}{X_{WW}}=\frac{X_{DW}}{\frac{B}{\delta_{DW,B}}+L_{DW}}$$ (22)

where *X*_*WW*_ is larval WW and *δ*_*DW*,*B*_ is the DW fraction of structural biomass.

### Collection of experimental data from literature

The literature was searched for information linking experimental data on larval DW or WW, lipid and protein content, CO_2_ production rate, and DW content, and development time. These data were used for a meta-analysis of the general growth patterns of BSF larvae to elucidate key variables to be included, and the broadness of larval performances, which should be covered by the growth model. The literature was furthermore searched for data combining kinetic and stoichiometric measurements on growing BSF larvae reared under different conditions and on different substrates, to allow the model to be tested against the broadest possible array of larval performances. Data were read from tables or if necessary, from graphical representations using the program Graphreader (www.graphreader.com). In some cases, data were recalculated into new representations because different papers present their results in various formats. All data collected or recalculated from literature are shown in S1 Table.

### Simulation of experimental data

The model was tested up against all the relevant sets of data combining kinetic and stoichiometric measurements on BSF larvae, identified during the literature search. Parameters and conversion factors, used in Eqs 1–22 were taken from literature when available, predicted from literature data, or estimated by fitting the growth model to experimental measurements as described below. The goodness of fit between model and measured data was evaluated as the average mean error, AME between model and measurement

$$\text{AME}=\frac{\left|z_{i,obs}-z_{i,model}\right|}{n_{i}}$$ (23)

where *z*_*i*,*obs*_ and *z*_*i*,*model*_ represent measured and modelled values of DW, lipid content, DW content, or CO_2_ production rate, respectively, and *n*_*i*_ is the number of measurements of each variable.

Due to differences in what data is available in different papers, the modelling procedure was adjusted according to the available data. In general, the model was fitted to data by using the solver function, build into Excel, to predict the smallest AME between modelled and measured DW and lipid content. If lipid content had not been measured in the paper, the model was fitted to predict the smallest AME between modelled and measured DW and CO_2_ production rate, and the ability of the model to then predict larval DW content was used to verify that model predictions were meaningful. Further details and exceptions regarding estimation of parameters and simulation of individual sets of data are described in Results and Discussion.

## Results and discussion

### General growth patterns

**Fig 2:**
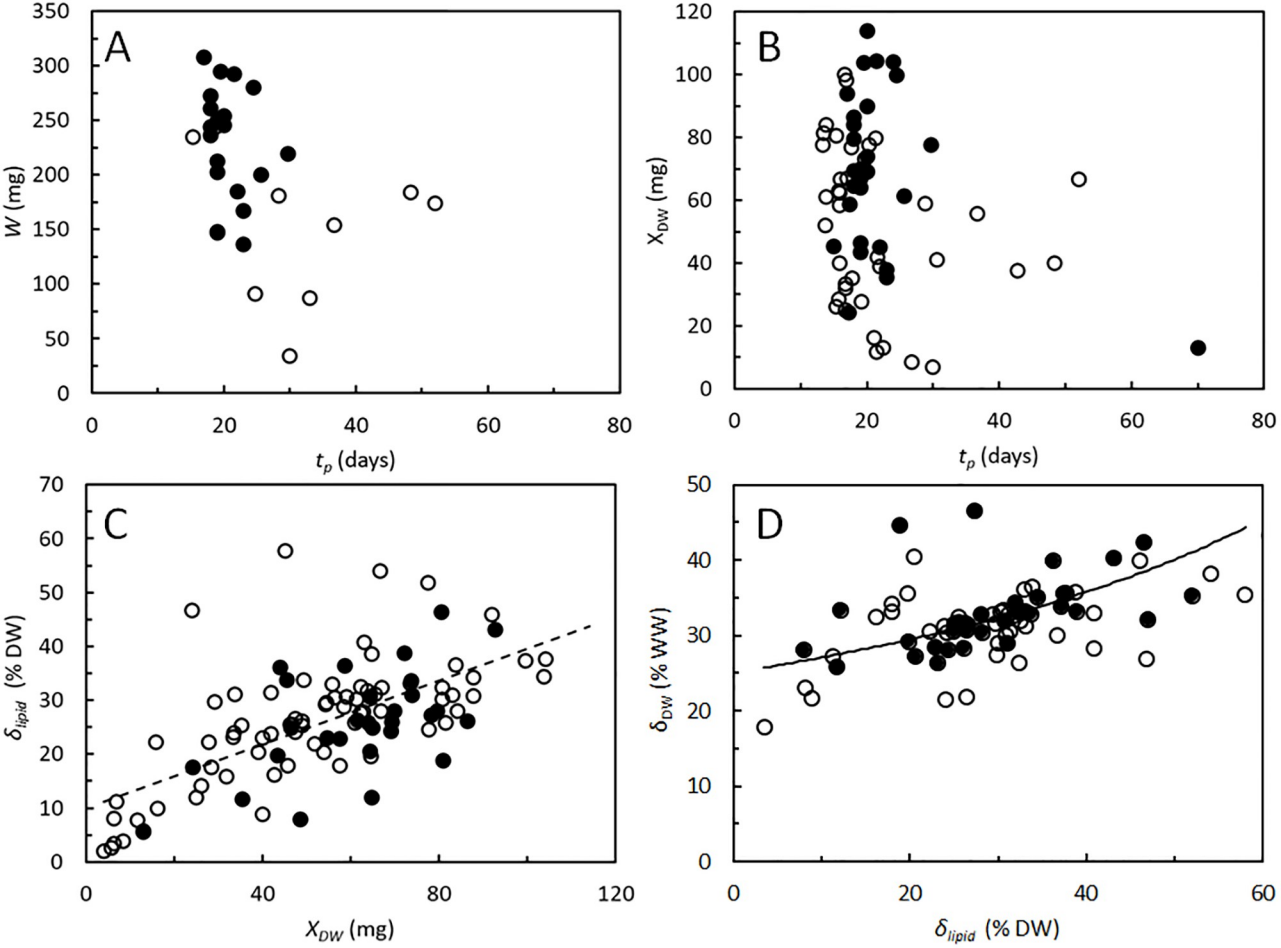
*Hermetia illucens*. A. Wet weight, *W* of full grown BSF larvae (○) or prepupae (●) *vs*. age, *t*_*p*_. B. Dry weight, *X*_*DW*_ of full grown BSF larvae (○) or prepupae (●) *vs*. age. C. Lipid content, *δ*_*lipid*_ of full grown BSF larvae (○) or prepupae (●) *vs*. dry weight. Intercept of best straight line indicates minimal lipid content of 10% DW. D. Dry weight content, *δ*_*DW*_ of full grown BSF larve (○) or prepupae (●) *vs*. lipid content. Curve modelled by Eq 22. Data collected from larval cultivations in various substrate compositions, different physical conditions, and different experimental protocols [7, 9, 10, 14, 21, 22, 25–27, 35–48]. Data in S1 Table.

General growth patterns of BSF larvae were examined using experimental data from literature. Fig 2A and 2B show WW and DW of BSF larvae and prepupae as function of the time it took for them to reach either the prepupal stage, *t*_*p*_ or their maximal weight. The maximal weight is reached shortly before the individuals can be identified as prepupae [9] and these larvae are here denoted full-grown. There is a tendency for the BSF larvae and prepupae to grow heavier the shorter their development time is. Fig 2C and 2D show that the larvae also tend to obtain higher lipid contents the heavier they become (*r*^2^ = 0.42), and their DW content is positively related to their lipid content. In contrast seems there to be no apparent relationships between protein content and DW (*r*^2^ = 0.09), protein content and lipid content (*r*^2^ = 0.04), nor ash content and DW (*r*^2^ = 0.07), see S1 Fig. It was due to these findings that storage lipids were partitioned into a separate variable in the growth model, while proteins and inorganics were included in the structural biomass (Fig 1). Lipids, proteins, and inorganics make up most of the larval DW. The average lipid content, based on measurements from 20 of the studies shown in Fig 2, was 27% ± 11% of the larval DW [7, 21, 22, 25–27, 35–47]. The average protein content was 48% ± 10% of larval DW based on 17 of the studies in Fig 2 [7, 21, 22, 25, 26, 36–43, 45–47], and the average ash content was 14% ± 10% of larval DW, based on 11 of the studies in Fig 2 [9, 14, 22, 39–43, 45–47]. Not surprisingly, Fig 2 and S1 Fig show considerable variability in the performance and composition of the BSF larvae as the figures combine results from a variety of substrates and a wide spectrum of rearing conditions.

### Estimation of parameters

Table 1 lists the parameters and variables that are included in the growth model, Eqs 1–22. Some parameters had to be estimated by fitting the model to measured data from individual cultures for the model to describe these data, while others were used universally, as described below.

**Table 1:** Variables and parameters used for modelling of growth and biomass composition in BSF larvae by Eqs 1–22.

Symbol	Description	Unit	Value	Source
Variables included in the growth model				
*A*	Weight of assimilates	mg	-	-
*B*	Weight of structural biomass	mg	Variable	-
*L*	Weight of lipids	mg	Variable	-
*I*	Instar	-	5–7	-
*a*	Specific assimilation rate	day^-1^	Variable	-
*μ*_*B*_	Specific growth rate of structural biomass	day^-1^	Variable	-
*B*_*max*_	Maximal weight of structural biomass	mg	Variable	-
*L*_*tot*_	Weight of total lipids	mg	Variable	-
*t*_*0*_	Larval age at beginning of experiment	day	Variable	-
*t*_*p*_	Age where larvae reach maximal weight and become prepupae	day	Variable	-
Rates included in the growth model				
*r*_*A*_	Assimilation rate	mg day^-1^	Variable	-
*r*_*B*_	Rate of production of structural biomass	mg day^-1^	Variable	-
*r*_*L*_	Rate of production of structural lipids	mg day^-1^	Variable	-
*r*_*CO2*,*m*_	Rate of CO_2_ production due to maintenance	mg day^-1^	Variable	-
*r*_*CO2*,*B*_	Rate of CO_2_ production due to synthesis of structural biomass	mg day^-1^	Variable	-
*r*_*CO2*,*L*_	Rate of CO_2_ production due to synthesis of structural lipids	mg day^-1^	Variable	-
Parameters used in the growth model				
*a*_*max*_	Maximal specific assimilation rate	day^-1^	0.25–1.4	This study
*B*_*max*,*0*_	Maximal weight of structural biomass at optimal conditions	mg	65–90	This study
*Y*_*B*_	Cost of growth of structural biomass	-	0.44*	[9]
*Y*_*L*_	Cost of synthesis of storage lipids	-	0.42	[50]
*m*	Specific maintenance rate	day^-1^	0.08*	[9]
*t*_*p*,*min*_	Minimal developmental time from neonate to prepupae	day	13	[11]
*ρ*	Rate by which *B*_*max*_ decreases in period after *t*_*p*,*min*_	mg day^-1^	1	This study
*Α*	Coefficient, adjusting specific assimilation rate	-	0.26–3.27	This study
*β*	Coefficient, adjusting specific growth rate	-	0.88–3.19	This study
*Δt*	Time interval used for numerical modeling	day	0.01–0.02	This study
Variables derived from the growth model				
*X*_*DW*_	Biomass dry weight	mg	Variable	-
*L*_*tot*,*DW*_	Lipid dry weight	mg	Variable	-
Ratios				
*δ*_*C*,*B*_	Carbon fraction of structural biomass	-	0.49	[51]
*δ*_*C*,*L*_	Carbon fraction of lipids (based on trilaurin)	-	0.79	-
*δ*_*C*,*CO2*_	Carbon fraction of CO_2_	-	0.27	-
*δ*_*L*,*B*_	Lipid fraction of structural biomass	-	0.1	Fig 2C
*δ*_*O*_	Organic fraction of larval DW	-	0.9	S1 Fig
*δ*_*DW*,*B*_	DW fraction of structural biomass	-	0,25	Fig 2D
*δ*_*DW*_	DW fraction of larval WW	-	Variable	-
*δ*_*lipid*_	Lipid fraction of DW	.	Variable	-
*δ*_*I*_	Weight ratio between succeeding moulting phases	-	5	[41, 52]

*Simulations in Fig 5 were carried out using different values

The maximal specific assimilation rate, *a*_*max*_ was estimated by fitting modelled DW to measured values during the part of the growth phase attributed to instar 1–5, where growth was close to being exponential. Graphical plots of ln-transformed DW *vs*. time were used to provide visual representations of the relationship between model and measurement. The maximal weight of structural biomass at optimal conditions, *B*_*max*,*0*_ is not a well-known quantity, but values of either 65 mg or 90 mg resulted in good fits between modelled and measured DW, and either of these values was used for simulation of individual growth curves, described below.

The cost of growth, *Y*_*G*_ = 0.44 and maintenance, *m* = 0.08 day^-1^ for whole larvae have been estimated on chicken feed [9], and here used also as cost of growth and maintenance of the structural part of the biomass. In cases, where experimental measurements were available, simulations were carried out using different cost of growth of structural biomass and maintenance, as discussed in relation to the individual experiments below. The cost of converting assimilates into lipids will depend on the substrate. BSF larvae can synthesize lipids *de novo* or take up lipids from the substrate [49]. In grain-based substrates, lipid synthesis is at the expense of carbohydrates where 240 carbon atoms from carbohydrate are transferred into CO_2_ and lipid at a molar carbon ratio of 72:168 [50]. This corresponds to *Y*_*L*_ = 0.42, which is the value used in this work.

It is highly variable, how long time BSF larvae need to complete their development from neonate to prepupae (Fig 2A and 2B) but can be as short at 13 days at optimal conditions [11]. Thus, in the model the minimal developmental time, *t*_*p*,*min*_ = 13 days. The rate, *ρ* by which *B*_*max*_ decreases in the period following *t*_*p*,*min*_ was set to 1 mg day^-1^, since this value provided good fits between modelled and measured DW.

The coefficient, *α* downregulating specific assimilation rate was estimated by fitting the Eq 17 to DW measurements. Secondly, the coefficient, *β* downregulating specific growth rate was estimated by fitting the Eq 18 to measured lipid contents or CO_2_ production rates, depending on which data were available.

The modelled was programmed to allow Eqs 17 and 18 to be evaluated numerically via 2,000 time intervals, *Δt*. For practical reasons were each time interval adjusted to 0.01–0.02 days (with no effect on results), to end simulations close to the time of the last experimental measurements.

Carbon is assumed to make up 49% of the organic fraction of structural biomass [51], why *δ*_*C*,*B*_ = 0.49. The carbon content of the lipids was estimated as the carbon content of trilaurin, *δ*_*C*,*L*_ = 0.74, since lauric acid is normally dominating the lipids of BSF larvae [35]. CO_2_ contains 27% carbon, i.e. *δ*_*C*,*CO2*_ = 0.27.

The lipid fraction of the structural biomass is likely close to the lowest lipid contents that are observed in BSF larvae, i.e. *δ*_*L*,*B*_ ≈ 0.1 [28]. This value agrees well with the regression line in Fig 2C, indicating that the smallest of the full-grown BSF larvae, expected to have only a minimum of lipids stored in reserve, also contained around 10% lipid at time of harvest. The organic fraction (volatile solids) of the structural biomass of BSF larvae is assumed to make up 90% of the DW, i.e. *δ*_*O*_ = 0.9. The median from 10 independent studies [9, 22, 39–43, 45–47] was 89%, with no apparent relationship to the DW of the full-grown larvae and prepupae (*r*^2^ = 0.06), see S1 Fig. BSF larvae contain 20–45% DW (Fig 2D). The DW content of the structural biomass should thus be in the lower end of this interval, and simulations were carried out with *δ*_*DW*,*B*_ = 0.25.

The expected weight ratio between two succeeding moulting phases depends on the total weight gain and number of instars. Neonate BSF larvae have DW’s of 0.02–0.03 mg [41, 52] and may grow above 100 mg [14]. Correspondingly, each instar thus increases four to fivefold in weight, and expertly, *δ*_I_ = 4–5. Best agreement between model and data was obtained with *δ*_*I*_ = 5, which was the value used in simulations of growth curves.

### Availability of data sets combining stoichiometric and kinetic measurements on BSF larvae

Four data sets, including 10 different rearing conditions, were identified in the literature, which allow the kinetics of biomass production to be related to lipid accumulation, CO_2_ production, or changes in DW content of growing BSF larvae. The growth model was tested against all 4 data sets. None of the available data sets include measurements of all variables and detailed kinetic data on lipid accumulation are available from just 2 growth experiments. DW content during growth was therefore included in the model as an indirect measure of larval lipid content (Fig 2D). One set of growth curves from BSF larvae fed different daily rations of substrate was also included to test the ability of the model to describe growth when feed is limited. In combination, the available data sets represent variable substrate compositions (though all are grain based) and rearing conditions and provides the opportunity to test the ability of the model to describe growth and lipid accumulation in various situations and to elucidate potentials and limitations to the model.

### Growth and lipid accumulation

**Fig 3:**
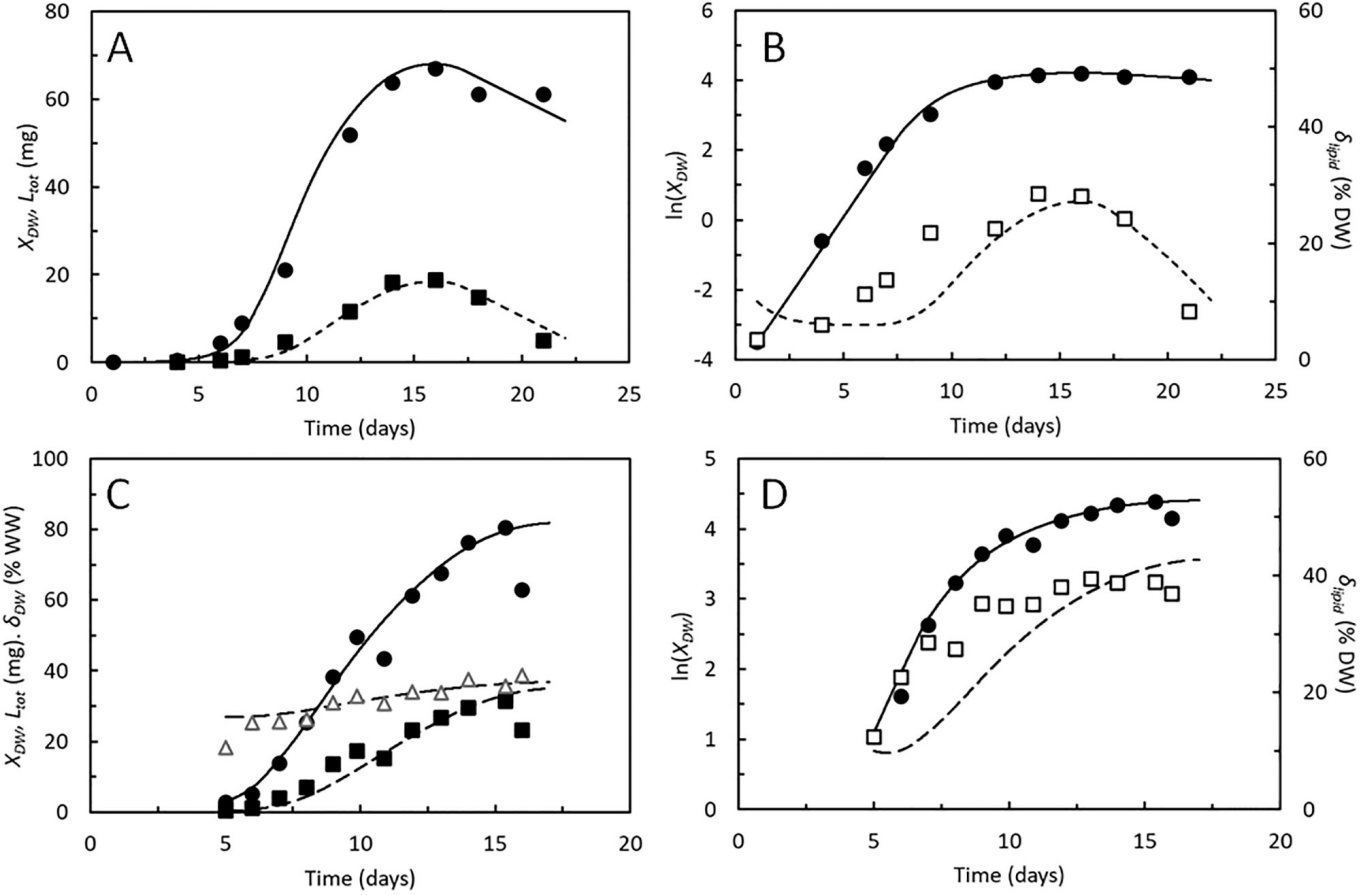
*Hermetia illucens*. A and C. Dry weight, *X*_*DW*_ (●), lipid content, *L*_*tot*_ (■), and dry weight fraction of biomass, *δ*_*DW*_ (△) of BSF larvae reared on chicken feed. B and D. Logarithmic transformed dry weight (●) and lipid to dry weight ratio, *δ*_*lipid*_ (□) of the BSF larvae. Data points from 2 independent studies [21, 22]. Curves predicted by the growth model. Parameters shown in Table 2.

Two independent studies [21, 22] link growth and lipid content of BSF larvae during most of their growth phase (Fig 3A and 3C). Both studies used chicken feed as substrate. The increase in DW is followed by an increase in lipid content. The lipid content decreases again when the larvae reach maximal weight and transform into prepupae. Measurements from this stage was, however, included only in one of these studies [21], during which the loss of lipids explained the loss of DW (Fig 3A). Proteins were not lost from these larvae when they transformed into prepupae. The growth model was able to describe the sigmoidal increase in biomass as well as the changes in lipid content in the larvae from both studies. All parameters used in simulations are shown in Table 2, as well as predicted developmental times, maximal specific growth rates of structural biomass, and peak values of DW, lipids, and DW content. Cost of growth and maintenance were adopted from [9], and a maximal weight of structural biomass of 65 mg gave good fits between model and data. The specific feed assimilation rates in the two experiments until the larvae reached the instar 6 stage (Eq 5), were estimated to 1.4 and 1.3 day^-1^, respectively. These values provided the best fit between modelled and measured DW of the larvae during the exponential growth phase, shown most clearly in Fig 3B and 3D, showing ln-transformed biomass DW, ln(*X*_*DW*_). Small larvae (instars 1–5) grew exponentially, seen from the linear increase of ln(*X*_*DW*_). The close relationships between model and measured DW’s show that the model predicts growth accurately.

**Table 2:** Parameters used for modelling of growth of BSF larvae shown in Figs 3–6, and selected variables predicted from the modelled results.

	Unit	Fig 3A	Fig 3B	Fig 4	Fig 4	Fig 4	Fig 4	Fig 4	Fig 5A	Fig 5B	Fig 5C	Fig 5D	Fig 6A	Fig 6B	Fig 6C	Fig 6D
Starter larvae																
*X*_*DW*,*0*_	mg	0.03	3	2.46	2.46	2.46	2.46	2.46	0.6	0.6	0.6	0.6	0.6	0.6	0.6	0.6
*Age*	day	1	5	5.8	5.8	5.8	5.8	5.8	5	5	5	5	5	5	5	5
Parameter																
*B*_*max*_	mg	65	65	90	90	90	90	90	90	90	90	90	90	90	90	90
*δ*_*C*,*B*_	-	0.49	0.49	0.49	0.49	0.49	0.49	0.49	0.49	0.49	0.49	0.49	0.49	0.49	0.49	0.49
*δ*_*C*,*L*_	-	0.79	0.79	0.79	0.79	0.79	0.79	0.79	0.79	0.79	0.79	0.79	0.79	0.79	0.79	0.79
*δ*_*L*,*B*_	-	0.1	0.1	0.1	0.1	0.1	0.1	0.1	0.1	0.1	0.1	0.1	0.1	0.1	0.1	0.1
*δ*_*O*_	-	0.9	0.9	0.9	0.9	0.9	0.9	0.9	0.9	0.9	0.9	0.9	0.9	0.9	0.9	0.9
*δ*_*DW*,*B*_	-	0.25	0.25	0.25	0.25	0.25	0.25	0.25	0.25	0.25	0.25	0.25	0.25	0.25	0.25	0.25
*δ*_*I*_	-	0.2	0.2	0.2	0.2	0.2	0.2	0.2	0.2	0.2	0.2	0.2	0.2	0.2	0.2	0.2
*a*_*max*_	day^-1^	1.4	1.4	1.21	1.06	0.60	0.34	0.25	1.2	1.2	1.3	1.4	1.1	1.1	0.9	0.8
*Y*_*B*_	-	0.44	0.44	0.44	0.44	0.44	0.44	0.44	0.44	0.59	0.63	0.76	0.44	0.44	0.44	0.44
*Y*_*L*_	-	0.42	0.42	0.42	0.42	0.42	0.42	0.42	0.42	0.42	0.42	0.42	0.42	0.42	0.42	0.42
*m*	day^-1^	0.08	0.08	0.08	0.08	0.08	0.08	0.08	0.08	0.09	0.15	0.23	0.08	0.08	0.08	0.08
*α*	-	0,92	1.02	1	1	1	1	1	0.81	0.48	0.26	0.28	1.19	0.92	3.27	2.59
*β*	-	2,02	1.01	1	1	1	1	1	1.17	0.94	0.90	0.88	3.19	1.66	1.38	1.13
*t*_*p*,*min*_	day	13	13	13	13	13	13	13	13	13	13	13	13	13	13	13
*ρ*	day^-1^	1	1	1	1	1	1	1	1	1	1	1	1	1	1	1
*Δt*	day	0.0105	0.006	0.02	0.02	0.02	0.02	0.02	0.012	0.012	0.012	0.012	0.01	0.01	0.01	0.01
Predicted variable																
*μ*_*B*,*max*_	day^-1^	0.92	0.92	0.79	0.68	0.36	0.18	0.12	0.78	0.70	0.71	0.66	0.71	0.71	0.57	0.50
*X*_*DW*,*max*_	mg	68	82	105	97	68	39	27	91	74	45	39	82	84	108	101
*t*_*p*_	day	16	13	20	21	27	34	37	21	25	26	25	18	20	20	22
*L*_*tot*,*max*_	% DW	27	43	41	39	32	24	20	35	35	26	25	18	27	39	41
*δ*_*DW*,*max*_	% WW	31	37	30	29	27	25	24	34	34	31	31	29	31	35	36
Goodness of fit (AME)																
*X*_*DW*_	mg	1.8	2.6	5.5	6.1	3.5	1.2	3.7	4.0	4.3	2.8	2.9	2.4	3.4	3.3	3.3
*L*_*tot*_	mg	1.0	2.1	-	-	-	-	-	-	-	-	-	-	-	-	
*δ*_*DW*_	% WW	-	1.5	-	-	-	-	-	2.6	3.6	3.6	5.5	4.9	2.1	1.5	2.0
*r*_*CO2*_	mg day^-1^	-	-	-	-	-	-	-	2,5	1.7	2.1	1.8	3.2	1.7	3.1	3.7

The coefficient *α* was close to 1 in simulations of both experiments, which is similar to the growth model by [18]. The coefficient *β*, giving the best fit between model and lipid content was 2 and 1, respectively, in the experiments in Fig 3A and 3C. It is this difference in *β* that make the model predict highest lipid content in the larvae in Fig 3C, indicating that production of structural biomass decreased most rapidly with weight in these larvae, leaving a larger fraction of the assimilates to be accumulated as storage lipids. Fig 3C also includes measured DW contents in the larvae. The model predicts an increase in this variable, based on the increase in larval lipid content, close to what was observed [22]. AME values of 1.8 and 1.0 mg between modelled and measured DW and lipid contents, respectively (Table 2), were small compared to the range of the measurements, 0–66 mg DW and 0–18 mg lipid, respectively, which also show that modelled values in general were close to measured ones.

Fig 3B and 3D also show the specific lipid contents of the BSF larvae, indicating that the lipid contents increased more rapidly in instar 1–5 than predicted by the model. Since these instars are responsible for just around 20% of the total weight gain in BSF larvae, underestimation of lipid accumulation in these stages will have only minor effects on the predicted lipid content in larvae approaching the prepupal stage where they will normally be harvested.

### Growth under feed limited conditions

**Fig 4:**
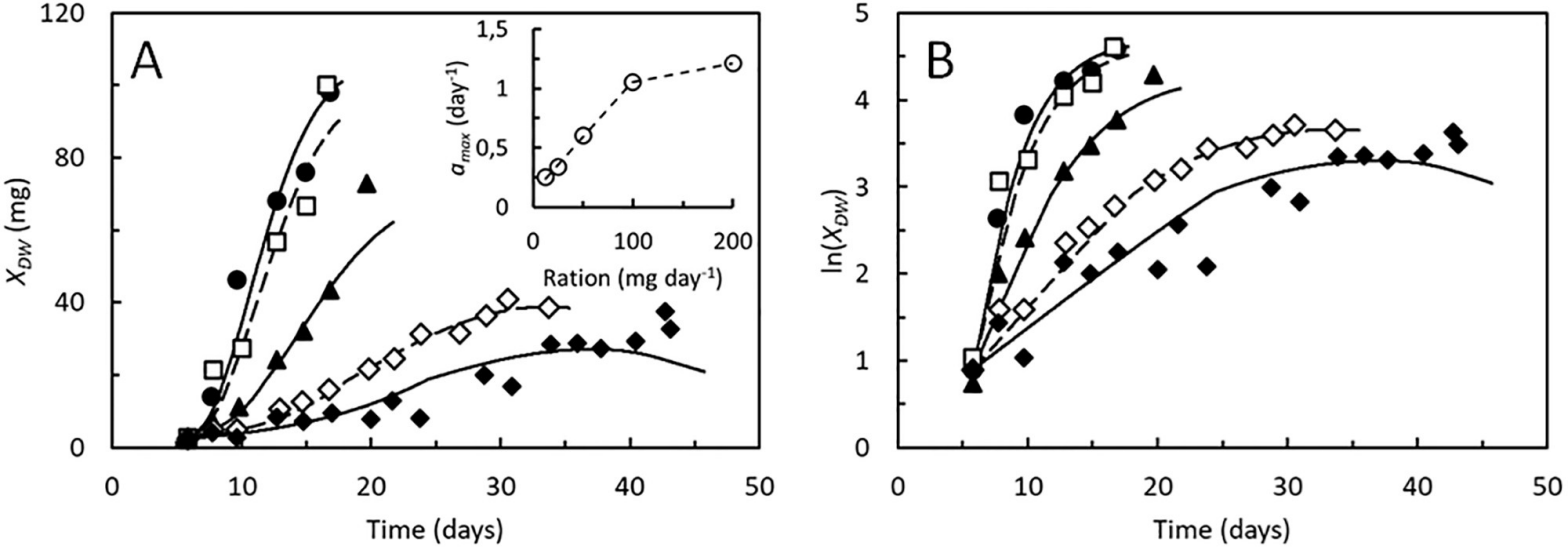
Hermetia illucens. A. Dry weight, *X*_*DW*_ and B. Logarithmic transformed dry weight of BSF larvae supplied a daily substrate ration of 12.5 (◆), 25 (◇), 50 (▲), 100 (□), or 200 (●) mg day^-1^ per individual [10]. Growth curves were modelled using identical parameters, except for the maximal specific feed up-take rate, which for each condition was estimated as the value giving the best fit to the data, resulting in *a*_*max*_ = 0.25, 0.34, 0.60, 1.06, and 1.21 day^-1^, respectively, for larvae fed the daily rations described above (inset A). Parameters shown in Table 2.

Feed limitation affects growth, demonstrated in a series of growth experiments by [10], in which BSF larvae were provided a daily substrate ration between 12.5 and 200 mg day^-1^ per individual (Fig 4). Low substrate rations restricted the rate of growth and the weight of the full-grown larvae. The dynamic growth model by [18] described well these experimental growth curves. The same is the case for the model presented here. The maximal feed assimilation rate (Eq 5) was for each feeding regime optimized to provide the best fit between model and DW measurements. In principle, the maximal feed assimilation rate was thereby made variable with respect to daily substrate ration. Other parameters were kept the same for all feeding regimes. Since only DW data is available from this study, it is not possible to make independent predictions of *α* and *β*, and both were set equal to 1 (Table 2). As result, the maximal feed assimilation rate was predicted to increase linearly with substrate ration up to 100 mg day^-1^ per individual. Above this ration, the BSF larvae seemed to have become feed sufficient (Fig 4A, insert).

### Larval performances on high and low-quality substrates

**Fig 5:**
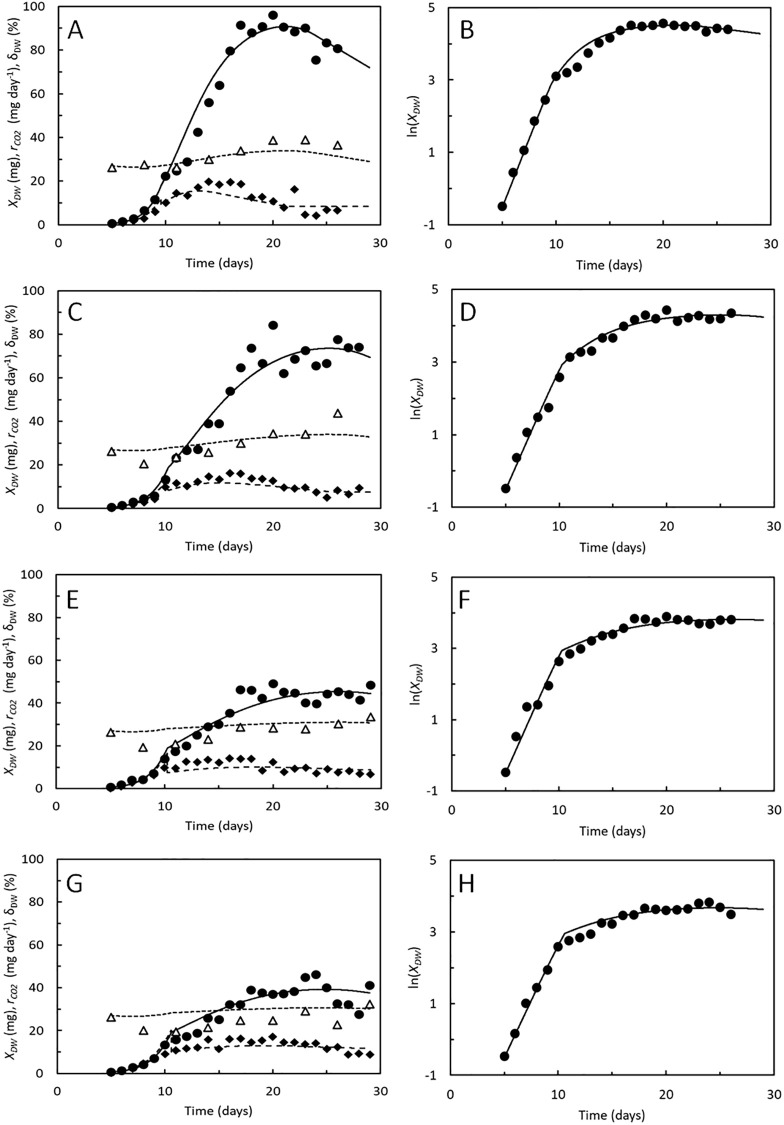
Hermetia illucens. A, C, E, G. Dry weight, *X*_*DW*_ (●), dry weight fraction of biomass, *δ*_*DW*_ (△), and CO_2_ production rate, *r*_*CO2*_ (◆) of BSF larvae reared on chicken feed (A), 75% chicken feed and 25% degassed sludge (B), 50% chicken feed and 50% degassed sludge (D), and 25% chicken feed and 75% degassed sludge (G). Logarithmic transformed dry weight (●) of the BSF larvae. Data points from [9]. Curves predicted by the growth model. Parameters shown in Table 2.

Measurements of growth, specific DW content, and respiratory CO_2_ production from BSF larvae published in [9] were used to evaluate the growth model across substrates of variable quality (Fig 5). The substrates were made from chicken feed (an excellent substrate) mixed with 0–75% degassed sludge (a poor substrate). Degassed sludge alone did not allow the BSF larvae to develop into prepupae. The cost of growth and maintenance were affected by the sludge content in the substrate.

Simulation of growth curves were carried out using the costs of growth and maintenance rates determined by [9], as shown in Table 2. Maximal specific assimilation rates of 1.2–1.4 day^-1^ resulted in best fits to the DW measurements during the exponential growth phase and were highest in the lowest quality substrates. Still, growth slowed, and the larvae became smaller the more degassed sludge was included in the substrate, since the higher cost of growth and maintenance resulted in increased CO_2_ production.

The coefficients, *α* and *β* were optimized to obtain the best fit (lowest AME) between model and measurements of DW and CO_2_ production. Most notable did *α* decrease from 0.81 in chicken feed to 0.28 when the sludge content was 75% (Table 2). This indicates that larvae in the instar 6 stage decreased feed assimilation more rapidly than explained by the logistic model when exposed to the degassed sludge. The specific DW content of the BSF larvae was then predicted with reasonable accuracy, particularly in the larvae reared on only chicken feed (Fig 4A) with no further optimization of parameters. The same was the case with respect to the CO_2_ production rate although the model indicated that CO_2_ production would peak earlier than observed. Fluxes of assimilates between the structural compartments shown in Fig 1 are thus predicted in accordance with measurements. Moulting is modelled as an instantaneous process. In reality, moulting delays larval development [34] and the largest discrepancies between modelled and measured larval DW’s and CO_2_ production rates are seen in the period where the larvae reach the instar 6 stage (Fig 4B, 4D, 4E and 4H). One cause for discrepancies between modelled and measured larval DW contents may be variable ash contents, but since no apparent relationship between ash content and other variables were identified (S1 Fig), the ash content is taken as a parameter rather than a variable in the model.

### Larval performances at different substrate moisture contents

**Fig 6:**
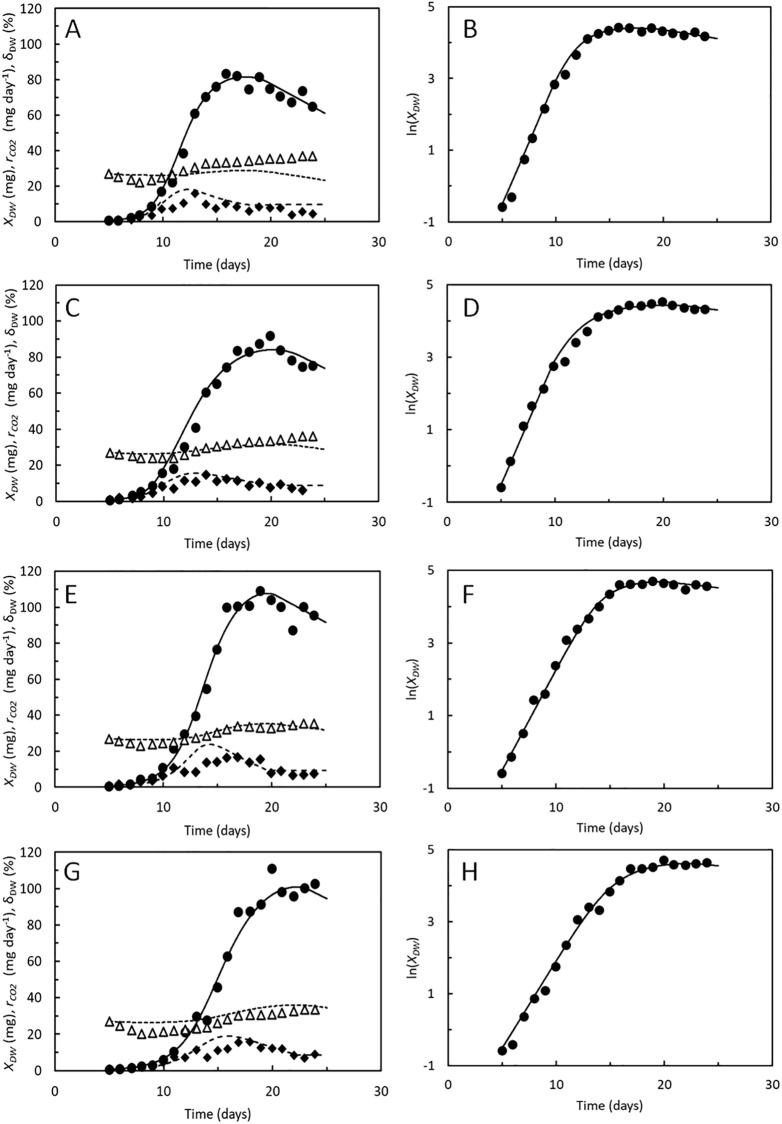
Hermetia illucens. A, C, E, G. Dry weight, *X*_*DW*_ (●), dry weight fraction of biomass, *δ*_*DW*_ (△), and CO_2_ production rate, *r*_*CO2*_ (◆) of BSF larvae reared on chicken feed with moisture contents of 45% (A), 55% (B), 65% (D), and 75% (G). Logarithmic transformed dry weight (●) of the BSF larvae. Data points from [14]. Curves predicted by the growth model. Parameters shown in Table 2.

The growth model was finally tested up against data from [14] on DW, specific DW content, and CO_2_ production from BSF larvae reared on chicken feed with controlled moisture contents of 45% - 75% (Fig 6). Increasing substrate moisture content resulted in larger larvae but lower specific growth rates while costs of growth and maintenance seemed unaffected. Substrate moisture content affects growth of BSF larvae [13, 18, 53–55] although the reasons behind this are not fully elucidated. Microbial activity played a major role in the experiments in [14] and affected the quality of the substrate within the first week of growth, particularly in the larval cultures with lowest substrate moisture contents.

Simulation of growth curves were carried out using the costs of growth and maintenance rates determined by [9]. Maximal specific assimilation rates decreased from 1.1 to 0.8 day^-1^ (Table 2) and *α* and *β* were also in this case optimized to provide the best fit between modelled and measured DW and CO_2_ production. Notably, the highest values of *α* of 2.6–3.3 was found in the two most moist substrates, indicating that the feed assimilation rates decreased less rapidly with larval weight, compared to the less moist substrates. This prolonged the growth period in the moist substrates. It is also notable that highest value of *β* was found in the substrate with lowest moisture content, indicating that the specific growth rate decreased slowest with larval weight at this rearing condition, and that these larvae thus accumulated the least amount of storage lipids. The shorter growth period and the comparatively low lipid accumulation meant that these larvae did not attain DW’s as high as those reared at higher substrate moisture content. The differences in *α* and *β* are in accordance with the suggestions in [14] that the BSF larvae grew fastest in the less moist substrates but microbial substrate degradation within the first week of cultivation hampered growth thereafter. This is thus an example where highest weight of full-grown larvae were not associated with shortest developmental time. The specific DW content of the BSF larvae was again predicted fairly accurately (Fig 5) with no further optimization of parameters. The same was the case with respect to the CO_2_ production rate, although the model again indicated that CO_2_ production would peak earlier than observed.

### Concluding remarks

The growth model presented in this paper is able to reproduce the data sets available in literature, which link kinetic data on growth to lipid accumulation in BSF larvae. The model predicts reasonably accurately also measured DW contents and rates of CO_2_ production (Figs 3, 5 and 6). Modelled growth curves are also in compliance with experimental measurements during feed limited growth (Fig 4) and AME values (Table 2) were in all cases small as compared to the range of the measurements. The model is thus able to describe performances of BSF larvae in a variety of situations despite the model provides a simplified representation of the metabolic processes of growing BSF larvae (Fig 1) and the fairly low number of experimental studies available for its development. The assumption that mainly lipids are re-metabolized to provide energy for maintenance is e.g., based on just one study [21], which is however supported by the uniform protein content of BSF larvae of different weights (S1 Fig). Other studies have shown that adult BSF’s weigh 3–4 times less than the prepupae [41]. Such large weight losses implies that other body components than lipids are also re-metabolized, at least after the prepupal stage. The present model is thus restricted to describe growth and development during parts of the BSF life history, from neonate larva to prepupa.

One purpose of the present model is to aid cross study comparisons of experimental results. The model fitted the growth curves in Fig 3 best with *B*_*max*_, the maximal weight of the structural biomass, at around 65 mg, while this parameter seemed closer to 90 mg in the larvae in Figs 4–6. This difference may suggest that the BSF strains used in these experiments differed in how large they grow, although most of the domesticated BSF populations seems to be genetically related [56]. It is also observed that *a*_*max*_, the maximal specific feed assimilation rate takes values from 1.1–1.4 day^-1^, unless the larvae were starved or stressed by high substrate moisture contents. These values likely represent the capacity for feed assimilation by BSF larvae, though specific feed assimilation rates have yet been quantified in just a narrow range of substrate types.

The present model resembles the dynamic model by [18] to some extent. Both models consider feed assimilation to be the overall rate limiting metabolic process and use a logistic function to reduce feed assimilation in relation to the weight of the larvae. In the present model, the assimilation rate is reduced only after the larvae have reached the instar 6 stage since growth of earlier instars are close to being exponential. The present model also reduces the specific growth rate of structural biomass by a second logistic function, affecting all instars. This reduces the need for assimilates for structural biomass synthesis below the rate of assimilation, generates an imbalance between feed assimilation and growth, and provides the surplus of assimilates, which are stored as lipids. It thus combines a kinetic model for uptake of feed and production of structural biomass with a differential energy budget model for accumulation and remobilization of storage lipids. The maximal weight of the structural biomass is made variable (Eq 16) to make the model dynamic for it to cover the wide variation of observed larval and prepupal weights (Fig 2), in combination with the opportunity to optimize the parameter for maximal feed assimilation rate, cost of growth, and maintenance. A weakness to both models, as well as to the static models used to analyze growth of BSF larvae [9, 14, 17], is that the logistic relationships between feed assimilation or growth are based on empirical findings and not directly supported by physiological processes. While the model by [18] allows feed assimilation to be a function of selected environmental variables (temperature, aeration rate, and substrate moisture content), the present model modulates specific feed uptake and growth rates by the coefficients *α* and *β*. The former approach could have been employed also in combination with the present model but depends on detailed knowledge on how the larvae respond to the environmental variables. Knowledge, which may not be available and be highly complex. The approach employed in the present model is therefore a practical solution although *α* and *β* have no direct functional links to the physiology of the larvae nor their environment. Still, determination of *α* and *β* by fitting the model to experimental data may provide useful information. In the example in Fig 5, the substrate dependent differences in *α* are likely physiological in origin (adverse effects from components in degassed sludge) while the moisture dependent differences of *α* and *β* in Fig 6 likely also reflect the effects of deteriorating substrate quality. The present growth model may thus be a useful tool for the analysis of growth and performance of BSF larvae under variable and complex rearing conditions. It can improve and deepen the analyses of data from growth experiments and provide insight into what causes the variability in performances of larvae of the BSF and possibly a wider range of insect species, when reared on different substrates and at different environmental conditions.

## Supporting information

S1 Data Dynamic growth model. The model programmed in Excel. Parameters can be modified (other functionalities are locked), and results visualized in ‘Main sheet’. The model itself, and quantitative results of modelling is available in the sheet named ‘Model’, columns E-U and columns W-AD, respectively.
(XLSX)

S1 Fig Protein, ash, and volatile solids in full-grown black soldier fly (*Hermetia illucens)* larvae and prepupae. A. Protein convent vs. dry weight, B. Protein content vs. lipid content, C. Ash content vs. dry weight, D. Volatile solids convent vs. dry weight in full-grown BSF larvae (○) or prepupae (●). Data in S1 Table.
(TIF)

S1 Table Biochemical composition of full-grown black soldier fly (*Hermetia illucens*) larvae and prepupae. Data collected from literature on developmental time, wet weight, dry weight, and contents of lipid, protein, ash, and volatile solids.
(XLSX)
