# Supplementary figures and images for: Dynamic modelling of feed assimilation, growth, lipid accumulation, and CO2 production in black soldier fly larvae
Source: PLoS One. 2022 Oct 26;17(10):e0276605. doi: 10.1371/journal.pone.0276605 (PMC9605037; doi:10.1371/journal.pone.0276605)

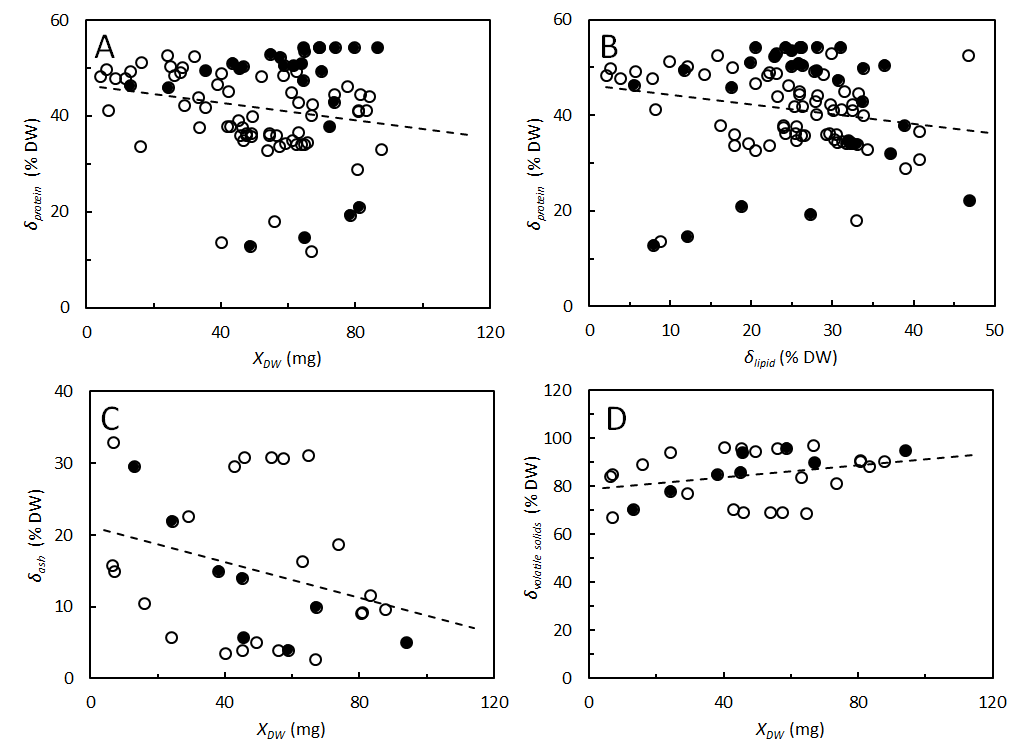

Supplement: S1 Fig — A. Protein convent vs. dry weight, B. Protein content vs. lipid content, C. Ash content vs. dry weight, D. Volatile solids convent vs. dry weight in full-grown BSF larvae (○) or prepupae (●). Data in S1 Table. (TIF) [file pone.0276605.s002.tif]
